# Role of Glutathione in the Regulation of Cisplatin Resistance in Cancer Chemotherapy

**DOI:** 10.1155/2010/430939

**Published:** 2010-09-14

**Authors:** Helen H. W. Chen, Macus Tien Kuo

**Affiliations:** ^1^Department of Radiation Oncology, Institute of Clinical Medicine, Medical College and Hospital, National Cheng Kung University, Tainan 70428, Taiwan; ^2^Department of Molecular Pathology, The University of Texas-MD Anderson Cancer Center, 7453 Fannin Boulevard, Houston, TX 77054, USA

## Abstract

Three mechanisms have been proposed for the role of glutathione (GSH) in regulating cisplatin (CDDP) sensitivities that affects its ultimate cell-killing ability: (i) GSH may serve as a cofactor in facilitating multidrug resistance protein 2- (MRP2-) mediated CDDP efflux in mammalian cells, since *MRP2*-transfected cells were shown to confer CDDP resistance; (ii) GSH may serve as a redox-regulating cytoprotector based on the observations that many CDDP-resistant cells overexpress GSH and *γ*-glutamylcysteine synthesis (*γ*-GCS), the rate-limiting enzyme for GSH biosynthesis; (iii) GSH may function as a copper (Cu) chelator. Elevated GSH expression depletes the cellular bioavailable Cu pool, resulting in upregulation of the high-affinity Cu transporter (hCtr1) which is also a CDDP transporter. This has been demonstrated that overexpression of GSH by transfection with *γ*-GCS conferred sensitization to CDDP toxicity. This review describes how these three models were developed and critically reviews their importance to overall CDDP cytotoxicity in cancer cell treatments.

## 1. Introduction

Cisplatin (CDDP) has been the mainstay for the treatment of a broad spectrum of human malignancies since it was approved by the FDA about 30 years ago. It has been used in the first-line treatment modalities of human malignancies, including testicular [1], ovarian [2, 3], cervical [4], bladder [5], head and neck [6], and small cell lung cancers (SCLCs) [3, 7, 8]. However, many patients eventually relapse and develop resistance to the treatment. Once patients develop resistance to platinum (Pt) drugs, other effective treatment options become limited. It is well known that CDDP acts on multiple cellular targets representing diverse signal transduction pathways. It is therefore conceivable that multiple mechanisms are involved in CDDP resistance, including reduction of drug transport and increased DNA adduct tolerance and repair [9, 10]. 

In this review, we focus on the role of glutathione (GSH) system in CDDP resistance. Three principal mechanisms are discussed here. The first mechanism involves the effects of GSH on the ATP-binding cassette (ABC) transporter-mediated CDDP transport; the second mechanism involves the redox-regulating capacity of GSH in detoxifying CDDP toxicity; the third mechanism involves regulation of the intracellular copper pool that affects CDDP uptake.

## 2. Role of GSH in ABC Transporter-Mediated CDDP Transport

The ABC transporters are a family of cytoplasmic membrane-spanned proteins that function as efflux pumps for eliminating antitumor agents, xenobiotics, and anionic lipophilic endogenous constituents [11–13]. Therefore, many of these transporters are also known as multidrug resistance proteins (MRPs). These transporters utilize ATP hydrolysis as energy source for substrate elimination [11]. Some transporters require GSH for substrate transport [11]. GSH may serve as substrate for conjugation reaction with CDDP prior to MRP-mediated transport. Ishikawa and Ali-Osman [14] first reported the formation of a Pt(GS)_2_ conjugate in L1210 leukemia cells. These investigators also reported that elimination of Pt(GS)_2_ across the membrane requires ATP, suggesting the involvement of an energy-dependent transporter of GSH-conjugate (termed GS-X pump) [15] in the elimination of Pt(GS)_2_ complex. Later, gene encoding MRP2, member ABCC2 in the ABC transporter family, was cloned; it was found that transfection of *MRP2* into HEK-293 cells conferred CDDP resistance (10-fold) in the transfected cells [16]. MRP2-mediated efflux requires GSH [17]. These results demonstrated that MRP2 is the GS-X pump for the elimination of CDDP. 

MRP2, like MRP1, can also transport GSSG itself, an oxidized form of GSH, with relatively higher affinity than does GSH [18, 19]. Thus, these ABC transporters can be considered as regulators of intracellular GSSG-GSH homeostasis and the associated redox maintenance (see below). Many studies have demonstrated direct interactions between GSH and ABC transporters [20, 21], suggesting that GSH may induce conformational changes that facilitate MRP-mediated substrate transport [11]. 

MRP2 is also known as a canalicular multispecific organic anion transporter (cMOAT) because of its high level of expression in the hepatic canalicular compartment and because it mediates the transport of a broad spectrum of nonbile salt organic anions from the liver into bile. cMOAT-deficient (TR-) Wistar rats are mutated in the gene encoding MRP2, leading to defective hepatobiliary transport of a whole range of substrates, including bilirubin glucuronide.

MRP2 mRNA and protein levels can be markedly induced by treatments with metalloid salts including sodium arsenite [As(III)] and potassium antimonyl tartrate in primary rat and human hepatocytes [22]. Expression of MRP2 in primary rat hepatocytes is also induced by CDDP [23]. In one study, a single subcutaneous injection of CDDP (5 mg/kg) into Male Sprague-Dawley rats resulted in >10-fold induction of MRP2 in renal brush-border membranes within one day of treatment whereas nonsignificant induction of MRP2 levels was found in the livers [24]. In normal rats, ~47% of the initial CDDP dose is excreted by the kidney whereas 1%–5% is excreted by the liver. The finding that increased expression of MRP2 in renal BBM upon injection of CDDP suggests that this transporter may be involved in the excretion of CDDP by the kidney. Since levels of MRP2 are already high in the hepatocytes, this may explain why only marginal increases of MRP2 was seen in the livers of CDDP-treated animals [24]. Moreover, a recent report showed that elevated MRP2 levels seemed to affect the efficacy of CDDP-based chemotherapy in hepatocellular carcinoma HCC [25]. 

 While Ishikawa and Ali-Osman [14] initially reported that formation of Pt(GS)_2_ complex reached a maximal level after 12 hrs in L1210 cells treated with 20 *μ*M CDDP, corresponding to ~60% of the intracellular Pt content, however, Berners-Price and Kuchel studied the reaction of CDDP with GSH inside intact red blood cells using ^1^H spin-echo nuclear magnetic resonance (NMR) and detected no formation of Pt-GSH bonds within 4 hrs of incubation [26]. Recently, Kasherman et al. [27] studied the interactions of CDDP with cell extracts prepared from ovarian cancer cells and found very little Pt(GS)_2_ by the [^1^H,^15^N]HSQC approach. Since CDDP can bind to many thiol-containing proteins and its primary cytotoxic target DNA, once inside the cells, these cellular constituents will compete against GSH for CDDP binding. These investigators found that the majority of glutathionated complexes were in fact in high molecular mass fraction (whereas the molecular mass of Pt(GS)_2_ is 809). Therefore, the significance of GSH-CDDP binding as an important step for CDDP elimination remains somewhat controversial.

## 3. Role of the GSH System as a Cytoprotector in CDDP Resistance

**Figure 1:**
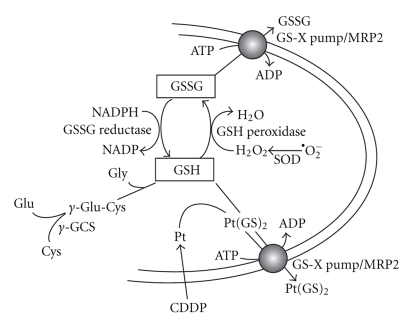
The role of GSH in MRP-mediated CDDP transport. *De novo *biosynthesis of GSH is carried out by *γ*-GCS, which conjugates glutamine (Glu) and cysteine (Cys) followed by GSH synthetase using glycine (Gly) as a substrate. GSH can be oxidized into GSSG by GSH peroxidase. SOD refers to superoxide dismutase. GSSG can be reduced to GSH by GSSG reductase. GSSG is a substrate of the MRP/GS-X pump whereas GSH functions as a cofactor for MRP-mediated CDDP transport.

GSH is an abundant thiol-containing tri-peptide (Glu-Cys-Gly), constituting 1∼10 mM in mammalian cells. *De novo* biosynthesis of GSH is controlled by the rate-limiting enzyme, glutamate-cysteine ligase (GCL, also known as *γ*-glutamylcysteine synthetase, *γ*-GCS) which consists of a catalytic (heavy) (*γ*-GCSh) and a regulatory (light) subunit (*γ*-GCSl). *γ*-GCS carries out the initial ligating reaction of glutamine (Glu) and cysteine (Cys). Production of GSH is accomplished by the subsequent reaction involving glycine (Gly) by GSH synthetase (Figure 1). GSH can be oxidized into GSSG by GSH peroxidase using H_2_O_2_ as a substrate whereas GSSG can be reduced back to GSH by GSSG reductase using NADPH as a cofactor. Therefore, GSH-GSSG system provides an important redox buffer in living organisms. 

As alluded to earlier, the transport activity of MRP2 (and other members of the MRP family) is regulated by GSH availability. Since *γ*-GCS is the rate-limiting enzyme for the biosynthesis of GSH, coregulation of *γ*-GCS and MRPs would facilitate the efflux activity (Figure 1). Indeed, we found that a number of cytotoxic agents can simultaneously induce the expression of both *γ*-GCSh and MRP, including CDDP [28], carcinogens [29], and prooxidants [30, 31]. Furthermore, enhanced expression of *γ*-GCSh/MRP1 was found in colorectal cancers, which are associated with inflammation-associated oxidative stress [32, 33]. These observations, taken together, strongly suggest that GSH/*γ*-GCS system is a molecular sensor of oxidative stress conditions [11].

### 3.1. Frequent Upregulation of GSH and *γ*-GCS in CDDP-Resistant Variants

Previous studies have reported that exposure of cultured cells to CDDP led to the development of CDDP resistance which was *correlated* with increased cellular GSH levels [34–39]. Moreover, GSH depletion by buthionine-sulfoximine (BSO) has been associated with increased sensitivity to CDDP [8, 14–17]. In many cases, when *γ*-GCS mRNA contents were measured, elevated levels of *γ*-GCSh mRNA were also correlated with CDDP resistance. These studies have been widely taken as that intracellular GSH levels play an important role in regulating CDDP resistance [34, 40–43], perhaps through a GSH-mediated cytoprotective mechanism. However, molecular mechanisms as how GSH functions as a cytoprotector for CDDP resistance have not been elucidated. 

However, this proposition has several caveats. One, these studies frequently used CDDP-treated cells and the observations were mostly correlative in nature. Whether elevated expression of GSH indeed is causally responsible for the CDDP resistance needs to be critically evaluated. Two, as alluded to above, induction of *γ*-GCS, and thus GSH, in CDDP-treated cells may be an oxidative stress-induced phenomenon because *γ*-GCS/GSH is a sensor/regulator of reactive oxygen species (ROSs) imbalance, and its expression can be induced by a wide array of cytotoxic insults. In many cases, elevated expression of *γ*-GCS/GSH under cytotoxic insult did not confer CDDP resistance in the treated cells [44]. Three, also as alluded above, normal cells usually contain *milli*molar levels of GSH. Stoichiometrically, these abundant amounts of thiol compound may already be sufficient to neutralize the cytotoxic effects of CDDP which are usually in *micro*molar ranges. Thus, increased GSH content found in most of CDDP-treated cells may not necessarily have a major impact in the detoxification mechanism of CDDP. As will be described below, we found that elevated expression GSH *per se* induces cellular sensitization to CDDP treatment [45].

### 3.2. Mechanisms of Upregulation of *γ*-GCS/GSH by Oxidative Stress

Both transcriptional regulation and posttranscriptional regulation have been reported for the upregulation of *γ*-GCSh by various cytotoxic assaults. Transcriptional regulation is mediated by an antioxidant response element (ARE, 5′-TGAGTCA) located at the promoter of the *γ* *-GCSh* allele, which interacts with the NF-E2-related transcription factor (Nrf2). Under unstressed conditions, majority of Nrf2 is in the cytosol and bound to Kelch-like ECH-associated protein (Keap1) which functions as a substrate adaptor for a Cullin-dependent E2 ubiquitin ligase complex and targets Nrf2 for ubiquitination and proteasomal degradation. Because Keap1 is a redox-sensitive E3 ligase, oxidative stress conditions induce Keap1 sulfhydryl group modification and conformational changes, resulting in Nrf2 release from proteasomal degradation and allowing it to translocate to the nucleus [46]. By heterodimerizing with the small Maf protein as coactivator, together, they bind to the ARE and transactivate *γ*-GCSh expression. Expression of MRP2 is also apparently regulated by Nrf2 signal. Livers from hepatocyte-specific *G* *c* *l* *c*/*γ*-*G* *C* *S* *h*-knockout mice showed elevated expression of Nrf2 and MRP2 due to elevated oxidative stress [47]. 

Regulation of *γ*-GCSh by oxidative stress is also controlled by posttranscriptional signal. We demonstrated that oxidative stress induces *γ*-GCSh mRNA stabilization through the p38 MAP kinase pathways. Under oxidative stress conditions, P38 MAP kinase activates MAPKAPK2, which promotes translocation of mRNA-stabilizing factor HuR from the nucleus to the cytoplasmic compartment, where it stabilizes *γ*-GCSh mRNA by binding to the AU-rich motif located at the 3′ untranslated region. The accumulated *γ*-GCSh mRNA produces elevated levels of GSH that feed back to suppress *γ*-GCSh mRNA stabilization [44] and the Nrf2 signal-mediated *γ*-GCSh transcription as mentioned above.

## 4. Role of GSH in Human High-Affinity Cu Transporter- (hCtr1-) Mediated CDDP Transport

In 1998, while we were studying the coregulation of *γ*-GCSh and MRP1 by CDDP, we found that transfection of recombinant DNA encoding the *γ* *-GCSh* subunit alone was sufficient to enhance GSH levels in the transfected cells [29]. Surprisingly, we also found that these stable *γ* *-GCSh*-transfected cells exhibited *hype*r*sensitivity *instead of resistance to the toxicity of CDDP. Hypersensitivity to the toxic effect of CDDP was associated with enhanced uptake of CDDP in these transfected cells. However, transporter for the uptake of CDDP was not available at the time. CDDP transporter was later identified as the high-affinity Cu transporter (hCtr1) [48] and the mechanism of this hypersensitization was due to the upregulation of hCtr1 in these transfected cells [45] (see below). 

### 4.1. Identification of hCtr1 as CDDP Transporter

Transporter for CDDP was identified using a genetic screening approach. Ishida et al. [48] mutagenized yeast cells with a transpose library after selecting mutants that were able to grow in the presence of a toxic dose of CDDP. One of the mutants was defective in the *Mac1* gene which encodes a copper concentration-dependent transcription factor for the expression of several genes involved in the uptake of iron and copper [49]. These investigators subsequently determined that yCtr1 is the target gene that could recapitulate the CDDP-resistance phenotype observed in the *m* *a* *c*1Δ mutant. Likewise, murine embryonic fibroblasts derived from *mCtr*1(−/−) animals exhibited reduced CDDP accumulation as compared with their respective wild-type counterparts. 

**Figure 2:**
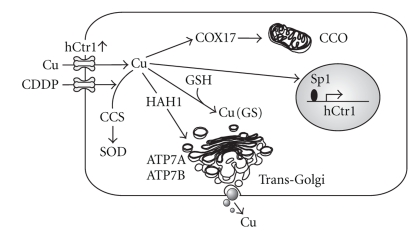
The effects of *γ*-GCSh overexpression on cellular Cu metabolism and CDDP transport. Overexpression of *γ*-GCSh, which catalyzes the ligation of cysteine (Cys) and glutamate (Glu), results in increased GSH levels. Excess GSH functions as a Cu depletor, as evidenced by the reduction in CCO and SOD activity, and holo-ceruloplasmin (Cu-Cpm) contents. Intracellular Cu deficiency upregulates hCtr1 expression, which is regulated by transcription factor, Sp1. Upregulation of hCtr1 enhances CDDP uptake, resulting in elevated sensitivities to CDDP treatment. CCS, HAH1, and COX17 are Cu chaperones that shuffle Cu to their respective targets as indicated by arrows.

The discovery that Ctr1 is involved in Pt drug transport underscores the important role of Cu metabolism in the efficacy of Pt drug chemotherapy. Copper is an essential micronutrient for cell survival, yet Cu overload is toxic. To meet their need for Cu while avoiding toxicity, all living organisms from yeast to humans have developed an evolutionarily conserved system to regulate Cu homeostasis. This system consists of a transporter (Ctr1) for Cu acquisition, chaperones (HAH1, COX17, and CCS) for Cu delivery to various intracellular compartments, and exporters (ATP7A and ATP7B) for Cu elimination (Figure 2). Although Cu entry can also be carried out by divalent metal transporter 1 (DMT1), which transports a broad range of divalent metal ions including Cu(II), the majority of Cu acquisition is accomplished by Ctr1, which transports Cu(I). The Belgrade rat, which has a mutation in *dmt1,* has no Cu-deficient phenotype [50]. Extracellular Cu exists in the oxidized form [Cu(II)] which is converted into Cu(I) by membrane-bound cupric reductases, relevant to the yeast Fre1 and Fre2 reductases [51, 52], for hCtr1-mediated transport.

Ctr1 plays an important role in the regulation of intracellular Cu homeostasis. This was first demonstrated in yeast. Expression of yeast *Ctr* (*yCtr1* and *yCtr3*) is transcriptionally upregulated under Cu-deplete conditions and is downregulated under Cu-replete conditions. During Cu-depleted conditions, the transcription factor Mac1p binds to the metal binding sequence [5′-TTTGC(T/G)C(A/G)] located at the promoters of *yCtr1* and *yCtr3* [53–55] and turns on the expression of these genes. Under Cu-replete conditions, Mac1 dissociates from the promoters, resulting in shut-down of the expression of *yCtr1* and *yCtr3.* In the meantime, the transcription factor Ace1 is activated to induce the expression of genes encoding Cu-chelating proteins (Cup1 and Crs5) and the antioxidant superoxide dismutase (SOD1) [56–58] to protect cells from Cu overload. Both Mac1 and Ace1 contain zinc finger (ZF) motifs that function as metallosensors. A transcriptional regulation mechanism is also involved in Cu(I)-dependent regulation of the *Drosophila CtrB* gene [59], a homologue of *yCtr1* and *yCtr3*. In addition to transcriptional regulation, posttranslational regulation has been reported for yCtr1 and yCtr3 proteins in response to Cu stress [60]. 

Available information in the literature regarding the mechanisms of regulation of human copper transporter (hCtr1) is controversial. It has been reported that regulation of mammalian Ctr1 is controlled at the posttranslational levels. Elevated Cu levels induce the trafficking of hCtr1 from the plasma membrane to endosomal/lysosomal compartments where Ctr1 is degraded [61, 62]. 

We investigated the regulation of hCtr1 expression in response to Cu concentration stress and found that levels of hCtr1 mRNA were decreased in cultured small cell lung cancer (SCLC) cells treated with CuSO_4_ but were increased in cells treated with the Cu-depleting agent bathocuproine disulfonic acid. We further demonstrated that the ZF transcription factor Sp1 plays an important role in the transcriptional regulation of hCtr1 [63]. These results, taken together, indicate that expression of hCtr1 is upregulated under copper deplete condition but is downregulated under copper replete conditions.

### 4.2. Mechanism of Sensitization of GSH-Overproducing Cells to CDDP Treatment

The findings that hCtr1 expression is regulated by intracellular Cu bioavailability prompted us to investigate whether sensitization of GSH-overproducing cells in the *γ* *-GCSh*-transfected cells to CDDP was due to reduced intracellular bioavailable Cu contents, resulting in upregulation of hCtr1 expression. It has been well established that Cu can form a Cu-GSH complex by directly interacting with its internal cysteine-SH residue of GSH. However, unlike formation of Pt(GS)_2_, formation of Cu(GS) is almost a spontaneous reaction and requires no enzymatic involvement [63–65], resulting in reduced intracellular bioavailability of Cu levels. We found that the *γ* *-GCSh*-transfected cells indeed showed Cu deficiency as evidenced by the reduced enzymatic activities of Cu, Zn superoxide dismutase (SOD1) and mitochondrial cytochrome C oxidase (CCO) as compared with those in the nontransfected cells [45]. Moreover, reduction of Cu content in ceruloplasmin, a copper-containing ferroxidase that plays an important role in mammalian iron homeostasis, was also found in the transfected cells. These enzymatic markers have been used as biochemical signature for intracellular Cu bioavailability [66]. Levels of hCtr1 expression in the three independently established *γ* *-GCSh*-transfected cells were 3- to 4-fold increases as compared with that in the parental cells. No substantial decreases in ATP7A or ATP7B levels were observed. Moreover, increased hCtr1 expression in these *γ* *-GCSh*-transfected cells was associated with enhanced CDDP uptake [45]. 

Sensitization of *γ* *-GCSh*-transfected cells to CDDP could be reversed when these cells were treated with GSH-depleting agent buthionine-sulfoximine (BSO). This reversal was associated with the reduced expression of hCtr1 due to the reinstated Cu bioavailability in the transfected cells as analyzed by activities of the Cu signature enzymes [45]. 

 Taken together, our finding demonstrated that elevated levels of GSH *per se* can sensitize CDDP toxicity. The elucidation that GSH functions as a Cu chelator in upregulating its transporter hCtr1 has important implications in cancer chemotherapy using platinum-based antitumor agents. We note that recent report has shown that another copper chelator, tetrathiomolybdate, can enhance CDDP sensitivity in ovarian cancer animal model [67].

## 5. Conclusion and Future Prospective

The three major mechanisms that control CDDP sensitivity by GSH described in the paper reflect the complexity of a small peptide that can regulate the efficacy of CDDP toxicity. The significance of each of these mechanisms may depend upon various cell types and/or different cell physiologic conditions. As alluded to above, CDDP can interact with many cellular targets and affect many signal transduction pathways to elicit its cytotoxicity. Likewise, GSH is an important redox regulator and redox signaling can affect many important cellular processes [11]. Thus, GSH may have far-reaching effects on CDDP sensitivity. Future investigations are needed to address the roles of GSH in the global effects of CDDP sensitivity.
